# Nutritional Modulation of Epigenetic Responses to Heat Stress in Farm Animals: Evidence, Limitations and Perspectives

**DOI:** 10.3390/ani16152369

**Published:** 2026-08-03

**Authors:** Ziyan Yang, Hongtao Liao, Yiyao Liu, Han Jiang, Xun Xiang, Kaiyang Liu, Liwei Guo, Yingbing Su, Xuejun Gao, Hanfei Wang, Niya Zhang

**Affiliations:** 1 College of Animal Science and Technology, Yangtze University, Jingzhou 434025, China; yangzy.stu@yangtzeu.edu.cn (Z.Y.); liaoht.stu@yangtzeu.edu.cn (H.L.); guolw@yangtzeu.edu.cn (L.G.); su-yingbing@163.com (Y.S.); gaoxj53901@163.com (X.G.); 2 National Reference Laboratory of Veterinary Drug Residues (HZAU) and MAO Key Laboratory for Detection of Veterinary Drug Residues, Huazhong Agricultural University, Wuhan 430070, China; liuyiyao@webmail.hzau.edu.cn; 3 Department of Animal Nutrition and Feed Science, College of Animal Science and Technology, Huazhong Agricultural University, Wuhan 430070, China; jianghan0828@163.com; 4 College of Veterinary Medicine, Yunnan Agricultural University, Kunming 650201, China; xiangxun2000@163.com; 5 Centre for Ecology and Conservation, Bioscience, University of Exeter, Penrvn Campus, Treliever Road, Penryn, Cornwall TR10 9FE, UK; k.liu3@exeter.ac.uk; 6 Center for Cross-Disciplinary in Animal Epidemic and Public Health, Hubei Jiangxia Laboratory, Wuhan 430200, China

**Keywords:** heat stress, nutritional intervention, epigenetics, methyl donor, short-chain fatty acid, precision nutrition

## Abstract

Rising global temperatures pose a serious threat to the productivity of farmed animals. Prolonged heat exposure impairs growth, reproduction and immunity; suppresses feed intake; and disrupts intestinal barriers, leading to substantial economic losses. Conventional cooling strategies offer limited relief, spurring research into the underlying molecular dysregulation. Epigenetic mechanisms link heat stress (HS) to gene expression. Specific nutrients that modulate these mechanisms could help mitigate HS. This review critically summarizes how methyl donors, short-chain fatty acids (SCFAs), vitamins, and phytochemicals reshape the epigenome of poultry, ruminants, and swine under HS conditions, focusing on heat shock protein (HSP) modulation, intestinal barrier reinforcement, and enhancement of antioxidant capacity. Across these interventions, several consistent observations emerge: methyl donors appear to modulate HSP70 expression, butyrate reinforces intestinal barrier integrity, and folate reverses stress-induced hypermethylation in swine—although direct epigenetic evidence remains limited in most cases. However, knowledge gaps remain, especially regarding aquatic animals and interactions among dietary components. By integrating mechanistic and empirical evidence, this review provides a basis for precision nutrition that goes beyond single-nutrient strategies. Targeted dietary intervention may influence thermal tolerance through epigenetic mechanisms. Looking ahead, direct measurements of epigenetic endpoints and systematic cross-species comparisons will be essential to validate and extend the current findings.

## Simple Summary

High temperatures pose a severe threat to the health of poultry, swine, and ruminants. Heat stress compromises animal physiological functions and growth performance, thereby resulting in substantial economic losses in the livestock industry. This review systematically summarizes dietary strategies for mitigating heat stress in farmed animals. Available evidence suggests that natural feed-derived nutrients can activate the endogenous protective systems of farmed animals. Dietary supplementation with such nutrients serves as a feasible and efficient approach to improving the thermotolerance of livestock. This nutritional regulation strategy presents promising application potential in modern livestock farming and facilitates the development of precision feeding regimens.

## 1. Introduction

The impact of global climate change is becoming increasingly evident [1]. The threats it poses to livestock and aquaculture are no longer long-term risks, but rather persistent challenges in reality [2]. This threat has not only incurred significant economic losses but also given rise to increasingly serious animal welfare issues [1,3]. Among these, the influence of heat stress (HS) is far more complex than merely affecting animal comfort [2]. It significantly inhibits animal growth, reproduction, and overall production performance [2], imposing a heavy economic burden on the industry [4,5]. Moreover, in a continuously high-temperature environment, animals struggle to maintain the stability of their physiological functions, and their welfare conditions are often affected as a result [6,7]. From a biological mechanism perspective, HS can trigger a series of systemic dysfunctions. It disrupts metabolic balance, causing animals to divert nutrients that should be used for growth to sustain survival. Meanwhile, it weakens the immune defense capacity and undermines the integrity of the barrier structure [2], ultimately leading to a systemic inflammatory response [8]. These multi-faceted effects not only reduce production efficiency and affect the quality of meat and milk but may also have an adverse impact on the development of offspring through in utero programming effects [9,10]. Although physical cooling and the renovation of farming facilities can alleviate HS to some extent, they fail to fundamentally address the molecular-level dysfunction. Therefore, the top priority now is to develop new approaches that target the core mechanisms of physiological resilience.

Behind these systemic effects, some conserved stress pathways are at play. The activation of the hypothalamic–pituitary–adrenal (HPA) axis, oxidative damage, and the transduction of inflammatory signals all play crucial roles [11,12]. These responses drive metabolic reprogramming, diverting energy from production performance towards maintaining the survival of the organism [12,13]. Epigenetics, including mechanisms such as DNA methylation, histone modifications, and non-coding RNA regulation, has emerged as an important link. It can translate environmental signals into lasting adaptive responses and even form heat memory [14,15,16].

Meanwhile, the concepts in nutrition are constantly evolving. Feed is no longer merely regarded as a source of nutrients. We are gradually realizing that it actually has the potential to “program” stress resilience [12,17]. Specific nutritional components such as methyl donors and bioactive substances of plant origin are believed to be able to shape the epigenetic landscape, thereby enhancing the adaptability of animals [12,18,19]. However, the current understanding is rather fragmented, and we still lack a unified mechanistic comprehension [20]. Many findings are limited to specific species, and there is insufficient translational evidence to link research with farming practices.

Despite the growing body of literature, several critical knowledge gaps remain. Most studies infer epigenetic involvement from changes in downstream gene expression, whereas direct measurements of DNA methylation, histone acetylation, or miRNA regulation are rarely performed in livestock. Systematic cross-species comparisons are also lacking, making it difficult to determine whether observed effects are species-specific or universally applicable. The potential synergistic or antagonistic interactions among dietary components have received almost no experimental attention, and research on aquatic species remains predominantly descriptive, with few nutritional intervention studies. These gaps collectively limit the translation of mechanistic insights into practical feeding strategies. The aim of this review is to clarify the complex interrelationships among nutrition, epigenetics, and HS in animals. To achieve this, we will first elucidate the physiological and epigenetic bases of HS, and then systematically evaluate the evidence for various nutritional intervention measures based on this. At the same time, we will also discuss the current challenges and future translational prospects, hoping to provide a relatively comprehensive perspective on this field. By integrating recent animal research and the latest advances in epigenetics, this work can lay a theoretical foundation for the formulation of precision nutrition strategies. These strategies can, on the one hand, help mitigate the losses caused by HS, and on the other hand, promote the sustainable development of farmed animals in the context of global warming.

## 2. Review Methodology

This review synthesises evidence on nutritional interventions that regulate HS responses in farmed animals (poultry, ruminants, swine and aquatic species) through epigenetic mechanisms. A structured literature search with systematic elements was conducted between September 2025 and November 2025 in PubMed, Web of Science, Scopus, and Google Scholar. Search terms combined the following keywords: “heat stress”, “thermal stress”, “epigenetic”, “DNA methylation”, “histone modification”, “microRNA”, “nutritional intervention”, “methyl donor”, “betaine”, “methionine”, “short-chain fatty acid”, “butyrate”, “folate”, “vitamin”, “phytochemical”, “polyphenol”, “poultry”, “broiler”, “ruminant”, “dairy cow”, “swine”, “piglet”, “aquatic”, “fish”. The search covered the last 15 years (2010–2026); earlier landmark publications were included when relevant.

Inclusion criteria were: (1) original research or reviews addressing epigenetic mechanisms (DNA methylation, histone modifications, non-coding RNAs) under HS; (2) clear description of nutritional interventions (single or combined) and their effects on epigenetic marks or HS-related phenotypes; (3) studies on farmed animals (poultry, ruminants, swine, aquatic) or relevant model organisms (as mechanistic reference). Exclusion criteria: non-English articles, non-peer-reviewed sources (e.g., conference abstracts, preprints), unavailable full texts, and production-only studies without epigenetic assessment. The initial search yielded approximately 380 records. Two reviewers (Z.Y. and H.L.) independently screened titles and abstracts against the inclusion criteria; disagreements were resolved through discussion with senior authors (H.W. and N.Z.). After deduplication and title/abstract screening, 210 articles remained. Full-text evaluation of these articles against the same criteria led to 101 finally included. Reference lists of included articles were manually screened by both reviewers, providing 25 additional papers.

Because of heterogeneity in experimental design, nutritional protocols, epigenetic assays, and species, a quantitative meta-analysis was not feasible. Evidence was synthesised thematically, grouped by animal category and intervention type. Key aspects extracted included epigenetic mechanisms, functional outcomes, and species-specific differences. No new animal experiments or primary data were generated, therefore ethical approval was not required.

During manuscript preparation, generative AI was used only to construct search strings and for language proofreading. All scientific content, terminology, and final versions of figures were independently reviewed, edited, and approved by the authors. No AI tools were used to generate or analyse primary research data cited in this review.

## 3. Biological Basis of Heat Stress and Epigenetic Response

### 3.1. Cascade Physiological Effects of Heat Stress

HS severely disrupts the physiological homeostasis of farmed animals, triggering a cascade of detrimental physiological reactions. The process initially involves a malfunction in thermoregulatory function, leading to hyperthermia. Subsequently, several pathways are activated. Although these pathways have certain adaptive effects, they often cause harm as well [21]. The most central response is the over-activation of the HPA axis, resulting in a substantial release of glucocorticoids [21], which further induces immunosuppression and metabolic disorders [21,22]. Meanwhile, the integrity of the intestinal barrier is compromised, intestinal permeability increases, endotoxin translocates, and this in turn triggers a systemic inflammatory response [21,23].

These disruptions, combined, impair key production performance. For breeding animals, reproductive capacity declines, and nutrient allocation shifts from growth to survival maintenance, resulting in slower weight gain and reduced feed efficiency [24,25]. In addition, the quality of livestock products such as milk, meat, and eggs deteriorates significantly [26,27,28]. Overall, HS is a multi-system pathological state that urgently requires comprehensive mitigation measures.

### 3.2. The Core Mechanism of Epigenetic Regulation of Heat Stress Response

**Figure 1:**
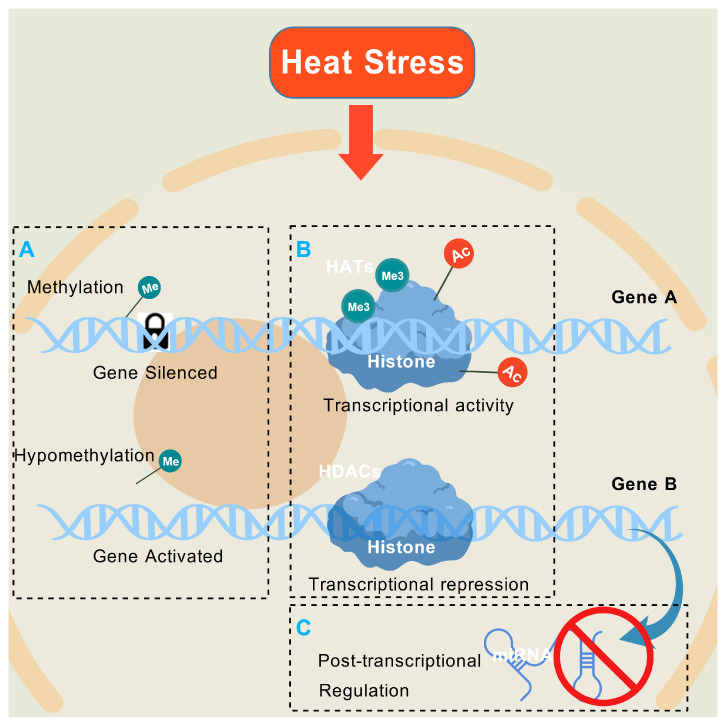
Core epigenetic mechanisms involved in the HS response in farm animals. HS triggers dynamic changes in three major epigenetic layers within the nucleus to fine-tune gene expression. (**A**) DNA methylation. Hypomethylation of promoters facilitates gene activation, while hypermethylation leads to gene silencing. (**B**) Histone modifications. Activation marks open chromatin for transcription, mediated by HATs. Repressive states are maintained by HDACs. (**C**) Non-coding RNAs. miRNAs bind to target mRNAs, leading to their degradation or translational inhibition, providing a rapid post-transcriptional regulatory layer. Together, these mechanisms orchestrate a tailored transcriptional response to thermal challenge.

Epigenetic regulation has emerged as a central mechanism by which organisms dynamically respond to environmental stresses such as high temperature. At the molecular level, HS induces profound changes in key epigenetic markers, including DNA methylation, histone modifications, and non-coding RNA expression. These changes collectively and precisely adjust the transcriptional program. On one hand, they may enhance survival ability, while on the other hand, under severe conditions, they may lead to physiological dysfunction (Figure 1). For instance, within the DNA methylation layer, the promoter region of the heat shock protein 70 (HSP70) gene undergoes dynamic methylation changes that modulate its transcriptional activity in response to heat challenge. In the non-coding RNA layer, microRNAs such as miR-21 are upregulated under heat stress and regulate apoptosis-related pathways. This process enables rapid and flexible responses without altering the underlying DNA sequence, thus establishing a molecular bridge between environmental challenges and gene expression.

Among these epigenetic modifications, DNA methylation is the most thoroughly studied. The promoter regions of key heat shock genes such as heat shock protein 70 (HSP70) undergo dynamic methylation changes under HS. Demethylation in this region usually activates the gene and enhances the cell’s protective function. In contrast, a high degree of methylation may silence genes essential for an effective stress response [29]. Besides DNA methylation, histone modifications also play a crucial role, which are mediated by enzymes such as histone acetyltransferases (HATs) and histone deacetylases (HDACs). These enzymes are highly sensitive to cellular stress signals and metabolic changes [30]. For instance, activation-related marks such as acetylation of lysine 27 on histone H3 (H3K27ac) and trimethylation of lysine 4 on histone H3 (H3K4me3) are enriched in the promoter regions of pro-inflammatory genes in the nuclear factor kappa B (NF-κB) pathway, thereby amplifying inflammatory signals under stress conditions [31,32]. Conversely, inhibitory marks can switch off some non-essential metabolic pathways to conserve energy [30]. Additionally, non-coding RNAs, especially microRNAs (miRNAs), are important post-transcriptional regulators in the heat-stress response. For example, miR-21 is typically upregulated under HS. It regulates apoptosis and autophagy processes by targeting key mRNAs in these pathways, thus influencing the cell’s life–death decision [33]. Other miRNAs finely modulate inflammatory and oxidative stress responses, adding a rapid and reversible layer of control to epigenetic regulation.

Overall, these epigenetic mechanisms jointly form an integrated regulatory network that can interpret the severity and duration of HS and then coordinate the activation and silencing of downstream genes. However, most of the current understanding is derived from in vitro experiments or model organisms, and there is a lack of mechanism validation in farmed animal species [33,34]. Elucidating the regulatory patterns of DNA methyltransferases (DNMTs), HDACs, and miRNA clusters under farming-related conditions is crucial for developing targeted epigenetic intervention measures and enhancing the heat tolerance of animals [35,36].

### 3.3. The Uniqueness of Epigenetics as an Environmental Sensor

Epigenetic mechanisms can be regarded as a highly sophisticated environmental sensor, enabling organisms to detect and respond to heat challenges with great precision and even record them. Unlike genetic changes, epigenetic modifications are dynamic and reversible. They not only maintain the integrity of the genome but also endow the organism with a flexible adaptive capacity [37]. This characteristic of being both stable and resettable determines its unique role in stress adaptation and memory formation across species.

The reversibility of epigenetic regulation is a crucial feature that contributes to the so-called “epigenetic stress memory”. Brief or non-lethal heat exposure can pre-adapt organisms to subsequent stress events, leading to faster and stronger responses. This memory is typically achieved through heritable chromatin changes, such as persistent histone modifications or DNA methylation patterns on key stress-responsive genes. These alterations persist even after the initial stress has subsided [38,39,40]. This pre-adaptation phenomenon has been observed in several species and is now considered a potential target for enhancing the heat tolerance of plants and animals [41,42].

In addition, epigenetic responses exhibit distinct tissue-specificity, which reflects the differences in physiological functions and susceptibilities of different tissues. For example, the liver may undergo epigenetic changes related to metabolic reprogramming and redox balance [43], while in the intestine, modifications associated with barrier integrity and inflammation regulation are commonly observed [44]. In the gonads, heat-induced epigenetic changes can affect gametogenesis and may even mediate the trans-generational inheritance of stress adaptation [29,45]. This tissue-specificity indicates the complexity of the epigenetic landscape and reminds us that future research requires organ-specific analyses.

Overall, the reversibility and tissue-specificity of epigenetic modifications jointly construct a unique layer of environmental sensing. These features not only enhance the adaptability of individuals but may also prepare future generations for upcoming climate challenges, which is attracting increasing attention in evolutionary biology and animal science [46,47]. Harnessing this plasticity offers promising prospects for formulating strategies to improve the stress resistance of important agricultural species.

## 4. Nutritional Intervention in the Core Pathways of Epigenetics

### 4.1. Molecular Mechanisms Linking Nutrition and Epigenetics

The interaction between nutrition and epigenetic regulation is a fundamental mechanism through which dietary components directly influence gene expression and cellular function. Specifically, certain nutrients and bioactive compounds act as molecular bridges. Instead of altering the DNA sequence, they exert their effects by regulating the activity of epigenetic enzymes and shaping the structure of chromatin [48]. This interaction between nutrition and the epigenome is particularly important under stress conditions, as metabolic demands and redox balance are disrupted at this time, providing an opportunity for targeted dietary strategies to restore physiological homeostasis.

**Figure 2:**
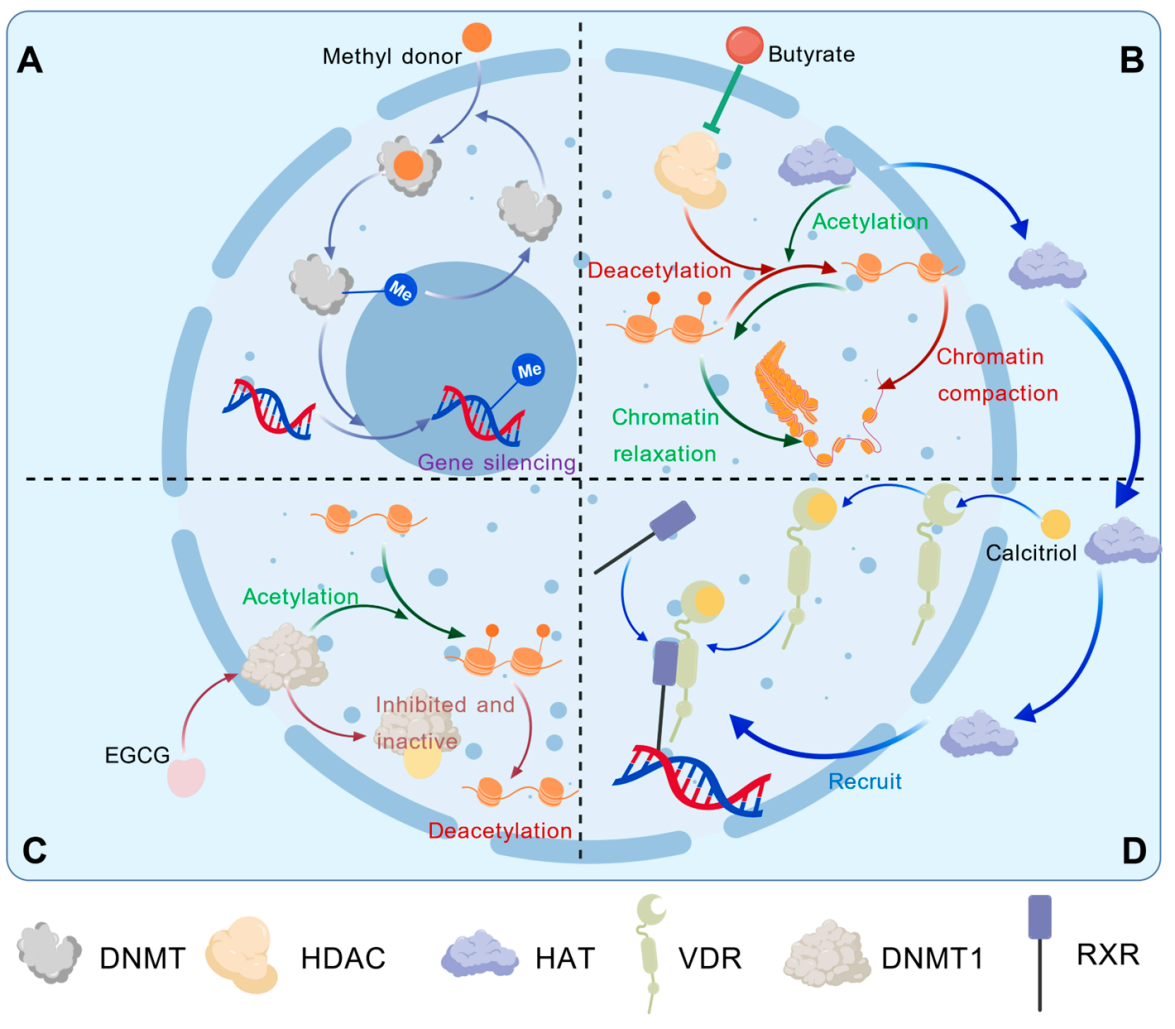
Molecular mechanisms of specific nutrients in regulating epigenetic responses to HS. Specific nutrients modulate the epigenome through distinct and targeted pathways to enhance thermal resilience. (**A**) Methyl donors serve as substrates for DNMTs, facilitating DNA methylation and transcriptional regulation of stress-responsive genes. (**B**) SCFAs like butyrate act as potent HDAC inhibitors. By blocking HDAC activity, butyrate promotes histone acetylation (Ac), leading to chromatin relaxation and activation of genes involved in cytoprotection and anti-inflammatory responses. (**C**) Phytogenic compounds (e.g., EGCG) directly bind to and inhibit enzymes like DNMT1, resulting in site-specific DNA demethylation and reactivation of silenced genes. (**D**) Receptor-mediated nutrients (e.g., calcitriol) exert epigenetic effects by binding to nuclear receptors (VDR/RXR). The ligand-receptor complex recruits co-activators with HAT activity to target gene promoters, facilitating chromatin opening and transcription. Abbreviations: DNMT, DNA methyltransferase; EGCG, epigallocatechin gallate; HDAC, histone deacetylase; HAT, histone acetyltransferase; RXR, retinoid X receptor; VDR, vitamin D receptor.

Methyl donors, such as betaine and S-adenosylmethionine (SAM), play a crucial role in this regulatory mechanism. They are important substrates for DNMTs (Figure 2A). By increasing the availability of methyl groups, these compounds can enhance the catalytic activity of DNMT and promote DNA methylation, especially in the gene promoter regions [49]. As a result, this can influence the transcriptional silencing or activation induced by environmental signals [49,50]. In addition to one-carbon metabolism, metabolites from both microorganisms and the host also act directly. SCFAs (such as butyric acid) produced by the fermentation of dietary fiber by gut microbiota are potent inhibitors of HDAC. They increase the level of histone acetylation and make the chromatin structure more relaxed (Figure 2B) [51]. Similarly, α-ketoglutarate, an intermediate of the tricarboxylic acid cycle, is a key co-factor for ten-eleven translocation (TET) enzymes. These enzymes are responsible for catalyzing DNA demethylation and are involved in dynamic epigenetic reprogramming [52,53]. Moreover, nutrients such as selenium and vitamin E can also indirectly affect epigenetic marks by regulating oxidative stress. They are important components of the antioxidant system and can reduce the excessive accumulation of ROS. Excessive reactive oxygen species (ROS) can promote abnormal histone modifications and global DNA hypomethylation [54,55]. By maintaining redox balance, these nutrients help maintain the accuracy of epigenetic regulation and support transcriptional patterns related to stress responses [56].

Overall, nutrients finely regulate the epigenetic state through multiple interconnected pathways, including direct substrate supply, enzyme activity regulation, and redox balance. Understanding these molecular bridges not only helps us understand how diet shapes phenotypic plasticity but also lays the foundation for implementing nutritional interventions through epigenetic mechanisms to improve stress resistance.

### 4.2. Epigenetic Modulation by Specific Nutrients

In addition to their traditional metabolic and antioxidant functions, specific nutrients such as vitamins, minerals, plant-derived compounds, functional amino acids, and microbiome-targeted supplements can actively shape the epigenome through a variety of sophisticated mechanisms [57,58]. Their ability to regulate the epigenetic modification system provides a viable approach to enhancing animals’ resistance to environmental stressors such as high temperature. These nutrients influence gene expression profiles either by directly interacting with epigenetic enzymes, altering the availability of co-factors, or triggering signaling cascades that lead to chromatin remodeling, thereby enabling a more robust transcriptional response when the organism is faced with heat challenges [59].

Among vitamins, vitamin D has drawn particular attention due to its genomic and non-genomic roles in epigenetic regulation. Once it binds to the vitamin D receptor (VDR), the receptor forms a heterodimer with the retinoic X receptor (RXR). This complex can recruit HATs such as p300/CBP to the promoter regions of target genes (Figure 2D). This process promotes histone acetylation, relaxes the chromatin structure, and enhances the transcription of genes related to anti-inflammatory and stress response pathways [60,61,62]. Notably, vitamin D deficiency is associated with hypermethylation of immune gene promoters, indicating its role in maintaining epigenetic flexibility under stress conditions [63].

Minerals such as zinc mainly maintain epigenetic stability through their structural and catalytic functions. The tertiary structure of zinc-finger (ZnF) proteins is zinc-ion dependent, and these proteins are important components of the functions of chromatin-remodeling complexes and transcription factors [64]. In the case of zinc deficiency, the activities of DNMTs and histone-modifying enzymes containing ZnF domains will be impaired, leading to abnormal gene silencing or activation [64]. Additionally, zinc is a co-factor for deacetylases, which are associated with stress resistance and longevity [65].

Bioactive compounds derived from plants, especially polyphenols such as epigallocatechin gallate (EGCG) in green tea, can serve as natural inhibitors of epigenetic enzymes (Figure 2C). EGCG can directly bind to DNA methyltransferase 1 (DNMT1) and inhibit its activity, resulting in site-specific DNA hypomethylation, which re-activates silenced tumor suppressor genes or stress-responsive genes [66,67]. Similarly, other polyphenols such as pterostilbene and curcumin can also regulate the activities of HDAC and HAT, delicately controlling the acetylation state to facilitate the expression of cell-protective genes [68,69,70].

Functional amino acids, such as arginine, can exert epigenetic effects through their metabolic by-products and signaling molecules. Arginine is a precursor of nitric oxide (NO), and NO can modify DNMTs and HDACs through S-nitrosylation, thus altering the activities of these enzymes [71]. In addition, the NO signaling pathway can also affect the expression of miRNAs, which regulate key processes in heat-stress responses such as apoptosis, autophagy, and inflammation [72,73,74].

Finally, prebiotics and probiotics regulate the host’s epigenetics mainly through microbial metabolites, especially SCFAs such as butyric acid, propionic acid, and acetic acid (Figure 2B). Butyric acid is a well-recognized inhibitor of HDAC. It can globally increase the level of histone acetylation and promote the expression of genes related to barrier integrity, immune regulation, and oxidative defense [75,76,77]. Probiotic strains can also influence the host’s miRNA profile and DNA methylation pattern, and may even mediate trans-generational epigenetic effects [78,79,80].

Overall, key nutrients act as potent epigenetic modifiers through multiple pathways, including enzyme inhibition, co-factor supply, receptor-mediated chromatin remodeling, and microbiome-mediated metabolite production. Their ability to induce dynamic and often reversible changes in the epigenome makes them ideal candidates for dietary intervention measures. These interventions aim to enhance animals’ heat tolerance and overall stress resistance in the face of climate change challenges (Table 1).

## 5. Empirical Insights into Nutritional Intervention-Mediated Epigenetic Regulation in Response to Heat Stress

In recent years, an increasing amount of practical evidence has emerged, demonstrating that nutritional interventions are indeed effective in alleviating HS in major livestock and poultry species, and epigenetic mechanisms are involved. These findings highlight that targeted dietary strategies can enhance heat tolerance and maintain production performance by regulating gene expression patterns without altering the underlying genetic code. However, the relevant results are often highly species-specific, which is closely related to differences in metabolism, digestive physiology, and the main production problems caused by high temperatures among different species.

### 5.1. Poultry: Methyl Donors and the Regulation of Heat Shock Protein Expression

In poultry research, the most direct evidence of nutritional regulation of heat-stress responses mainly comes from studies on methyl donors, namely betaine and methionine (Table 2). These compounds play a crucial role in promoting the synthesis of SAM, which provides methyl groups for DNMTs. Several studies have found that under cyclic HS conditions, supplementation with betaine or methionine can significantly affect the expression of HSP70 in the intestine and liver [83,84,85]. For instance, when 1000 mg/kg of betaine is added to broiler chicken feed, the mRNA level of HSP70 decreases, while the integrity of the intestinal barrier is improved, as suggested by the up-regulation of genes such as occludin, zonula occludens-1 (ZO-1), and claudin-1. At the same time, the levels of corticosterone and interleukin-1β (IL-1β) in the serum also decrease [85]. Similarly, the source of methionine is also important. Replacing DL-methionine with methionine-hydroxy analog (MHA) can not only enhance the expression of HSP70 in the liver and hypothalamus of broiler chickens but also improve antioxidant capacity, as reflected by increases in total antioxidant capacity (TAC) and glutathione peroxidase (GPx) [84]. In Peking ducks, supplementation with 0.35% methionine leads to the up-regulation of HSP70 in the duodenum, jejunum, and ileum, suggesting a dose-dependent epigenetic regulatory mechanism [83]. In addition, supplementation with chromium-methionine has also been indicated to reduce the expression of HSP70 in heat-stressed broiler chickens while increasing the expression of insulin-like growth factor 1 (IGF-1) [86].

It should be noted, however, that none of these studies directly measured epigenetic endpoints such as promoter methylation, histone acetylation, or miRNA profiles. Although changes in HSP70 expression are thought to reflect epigenetic regulation, this interpretation remains inferential without direct verification via bisulfite sequencing or ChIP-PCR. Collectively, the evidence is suggestive but inconclusive; the conclusions in this section should therefore be read as hypotheses requiring experimental validation.

### 5.2. Ruminants: Short-Chain Fatty Acids as HDAC Inhibitors in the Gut

In ruminants, the most compelling evidence of nutritional epigenetic regulation lies in SCFAs, especially butyric acid, which is produced by the fermentation of dietary fiber by microorganisms in the rumen and hindgut (Table 2) [87,88]. Unlike methyl donors that act on DNA methylation, butyric acid mainly functions as a potent inhibitor of HDAC in cell culture and rodent models. However, direct in vivo evidence for HDAC inhibition in ruminants under heat stress conditions has not yet been demonstrated [89]. This mechanistic distinction is important: unlike methyl donors, SCFAs do not provide methyl groups but directly reshape chromatin structure, enabling relatively rapid transcriptional reprogramming during heat challenges [87,88].

The functional significance of butyric acid-mediated HDAC inhibition is mainly reflected in the integrity of the intestinal barrier. HS can disrupt the tight junctions of the intestinal epithelium, leading to the translocation of lipopolysaccharide (LPS) and triggering systemic inflammation, which is a key factor contributing to the decrease in milk production and feed efficiency in dairy cattle [45]. Studies have found that supplementation with encapsulated sodium butyrate (20 to 40 g per head per day) can up-regulate the expression of tight-junction proteins (claudin-1, occludin, ZO-1) and antioxidant enzymes (SOD, GPx) in the jejunal and colonic epithelium of heat-stressed ruminants [87,88]. Mechanistically, these effects are hypothesized to be mediated by butyric acid inducing histone acetylation in the promoter regions of the above-mentioned protective genes. However, to date no study has directly verified this through chromatin immunoprecipitation (ChIP) in livestock.

Another idea worth mentioning is the supplementation of live *Saccharomyces cerevisiae*. This strategy can alter the rumen fermentation pattern, thereby increasing the production of endogenous SCFAs [87,88]. This method is associated with a decrease in inflammatory markers and an improvement in intestinal morphology under HS conditions, suggesting that increasing the availability of butyric acid by regulating the gut microbiome may be a viable alternative to direct butyric acid supplementation. That being said, the existing research has a significant shortcoming, there are too few direct measurements of epigenetic endpoints. Most studies infer HDAC inhibition from functional results, such as increased tight junction protein expression, rather than confirming it by quantitatively detecting acetylated histones or directly measuring HDAC activity. In addition, under HS conditions, whether butyric acid has a similar HDAC-inhibiting effect on tissues other than the intestine, such as the mammary gland and liver, remains unclear. In summary, while the HDAC-inhibitory properties of butyrate are well-established in non-ruminant models, the extrapolation to HS ruminants remains an inference that awaits direct molecular confirmation. Future studies should prioritize measuring histone acetylation status and HDAC activity in target tissues.

### 5.3. Swine: Folate and One-Carbon Metabolism in Gut Epigenetic Remodelling

In swine, research on nutritional epigenetics mainly focuses on folate and the one-carbon metabolism pathway. This pathway provides the methyl groups required for DNA methylation by synthesizing SAM (Table 2) [33]. Weaned piglets are a valuable research model as they are particularly sensitive to stress-induced intestinal dysfunction. HS exacerbates various post-weaning challenges, leading to villus atrophy, poor nutrient absorption, and growth inhibition [90].

The mechanism behind folate intervention is relatively well-understood. Folate deficiency can either lead to global DNA hypomethylation or, depending on the specific cellular environment and the duration of stress, result in hypermethylation in the promoter regions of certain key genes [33]. Under HS conditions, the metabolic demand for methyl donors increases. If the dietary supply cannot keep up, abnormal DNA methylation patterns will occur, silencing genes responsible for mucosal repair and nutrient transport. Studies on weaned piglets have indicated that supplementing with folate (2 to 3 times the NRC recommended amount) can reverse HS-induced hypermethylation, which occurs in genes encoding nutrient transporters (such as SGLT1, PEPT1) and tight-junction proteins, thus restoring the structure and digestive function of the villi [33,90].

There is also an emerging synergistic strategy, which is to use folate in combination with specific probiotic strains, especially *Lactobacillus plantarum* and *Lactobacillus rhamnosus*. These strains can synthesize folate in situ in the intestine [33]. The contribution of microorganisms to the host’s methyl donor pool provides a “dual-source” epigenetic support pathway, potentially reducing the amount of folate added to the feed. In addition, probiotics themselves can also affect the host’s epigenome by producing SCFAs (inhibiting HDAC) or regulating the expression profile of miRNAs, forming multi-level epigenetic effects. This area is currently in the initial exploration stage [90].

Despite these promising findings, significant knowledge gaps still exist. Most porcine-related studies have only detected the global DNA methylation level, for example, through LUMA or ELISA-based methods, without examining site-specific methylation changes in the promoter regions of relevant genes. There is a great need for whole-genome bisulfite sequencing (WGBS) studies under HS conditions to accurately identify which genes have undergone epigenetic dysregulation and whether folate supplementation truly reverses these specific markers [89]. In addition, under heat challenge conditions, the effects of folate on other epigenetic aspects such as histone modifications and non-coding RNAs have not been studied in swine yet. Collectively, while folate supplementation shows promising associations with improved gut methylation status and growth performance in weaned piglets, the evidence remains largely correlational. Most studies have relied on global methylation assays rather than locus-specific analyses, and none have integrated histone modification or miRNA profiling to provide a more complete picture of epigenetic regulation. Direct, mechanistic studies—particularly those employing whole-genome bisulfite sequencing or targeted chromatin analysis—are urgently needed to confirm whether folate truly reverses specific hypermethylation events at nutritionally relevant gene promoters, or whether the observed effects are mediated through alternative pathways.

### 5.4. Aquatic Animals: Unveiling the Link Between Non-Coding RNA Networks and Nutritional Epigenetics in Heat Stress

Research on HS in aquatic species has generated relatively detailed maps of heat-responsive miRNAs, long non-coding RNAs, and DNA methylation patterns. Studies in rainbow trout (*Oncorhynchus mykiss*), for instance, have revealed complex ceRNA networks in the liver under heat stress, while work in spotted sea bass (*Lateolabrax maculatus*) has identified muscle-specific miRNA-mRNA regulatory pathways [91,92]. Yet despite this descriptive richness, hardly any studies have investigated whether specific nutrients can actively reshape these epigenetic marks to improve heat tolerance [91,92,93]. In the few existing nutritional studies, such as the use of nano-selenium to reduce HSP70 transcription in catfish or the use of spirulina-coenzyme Q10 nanoemulsion to reduce HSP70 expression in tilapia, only the expression of downstream genes was detected, and no epigenetic end-point indicators were evaluated, whether they were DNA methylation, histone acetylation, or miRNA regulation (Table 2) [94,95]. A notable exception is that in Atlantic cod embryos under acute HS, the expression of one-carbon metabolism genes (MTHFR, MTR) and DNMTs (DNMT1, DNMT3A) changed, suggesting a possible direct association between heat challenges and the availability of methyl donors [96]. However, no feeding trials have yet attempted to counteract this effect by supplementing with methyl donors. Therefore, the literature on aquaculture is mainly descriptive, with few intervention-based studies. The potential of precision nutrition in alleviating HS in fish and shellfish has remained largely unexplored.

**Table 2:** Summary of key nutritional interventions, their epigenetic mechanisms, and functional outcomes in mitigating HS in livestock.

Nutrient Category	Specific Examples	Epigenetic Mechanism	Direct Epigenetic Evidence?	Evidence Strength	Key Functional Outcomes	Primary Species Studied	Refs.
Methyl donors	Betaine, Choline, Methionine	Provides methyl groups for SAM synthesis; Promotes DNA hypomethylation of HSP gene promoters.	No—methylation not directly measured; inferred from HSP70 expression	Speculative	Enhanced HSP expression; Improved growth rate and feed efficiency; Better laying performance.	Poultry	[26,51,52]
SCFAs	Butyrate	Potent inhibitor of HDACs; Increases global histone acetylation.	Partial—HDAC inhibition demonstrated in cell models; No direct measurement in ruminants under HS	Moderate	Enhanced expression of antioxidant genes and tight junction proteins; Improved gut barrier integrity; Reduced systemic inflammation.	Ruminants (Dairy cattle)	[53,77,78,79,88,97]
B-vitamins	Folate	Integral to one-carbon metabolism; supplies methyl groups for DNA methylation; Reverses stress-induced hypermethylation in the gut.	Partial—global methylation measured; locus-specific methylation not verified	Moderate	Maintained villus architecture and gut digestive function; Improved growth performance post-weaning.	Swine	[81,98,99]
Vitamins	Vitamin D (Calcitriol)	Binds to VDR/RXR complex; Recruits HATs to target gene promoters.	Yes—VDR-mediated HAT recruitment directly demonstrated	Strong	Enhanced transcription of anti-inflammatory and stress-responsive genes; Maintained epigenetic flexibility.	General/Multiple	[62,63,64,65]
Minerals	Zinc	Structural component of Zinc finger (ZnF) proteins in DNMTs and chromatin complexes; Cofactor for sirtuins.	Partial—structural role established; direct HS evidence lacking	Moderate	Maintained epigenetic enzyme stability and activity; Linked to stress resistance and longevity.	General/Multiple	[66,67]
Phytogenic compounds	Epigallocatechin gallate (EGCG)	Direct binding to and inhibition of DNA methyltransferase 1 (DNMT1); Causes site-specific DNA demethylation.	Yes—direct DNMT1 inhibition demonstrated in biochemical assays	Strong	Reactivation of silenced stress-responsive or tumour suppressor genes.	General/Multiple	[68,69]
	Pterostilbene, Curcumin	Modulates activity of HDACs and HATs.	Partial—enzyme modulation shown in cell models	Moderate	Fine-tunes acetylation landscape for cytoprotective gene expression.	General/Multiple	[70,72,73]
Functional amino acids	Arginine	Precursor for nitric oxide (NO); S-nitrosylates DNMTs and HDACs, altering their activity.	Partial—mechanism demonstrated in cell models	Moderate	Alters epigenetic enzyme function; Influences miRNA regulating apoptosis and inflammation.	General/Multiple	[74,75,76]
Microbiome-targeted	Probiotics (e.g., *Lactobacillus* spp., *S. cerevisiae*)	Modulates host epigenome via microbial metabolites; Influences host miRNA and DNA methylation patterns.	Partial—shown in model organisms; livestock data limited	Moderate	Enhanced gut health and folate status; Synergistic effects with dietary nutrients; Supports robust gut epigenetic response.	Poultry, Swine, Ruminants	[80,82,87,88,97]
Minerals	Selenium nanoparticles (Se-NPs)	Not measured (indirect via ROS reduction inferred)	No—only downstream gene expression measured	Speculative	Downregulates *HSP70*, *Caspase 3a*, *iNOS*; reduces apoptosis under HS	Giant catfish (*Pangasianodon hypophthalmus*)	[95]
Phytogenic/Nutraceutical	Spirulina + Coenzyme Q10 nanoemulsion (SCN)	Not measured (antioxidant-mediated protection inferred)	No—only downstream gene expression measured	Speculative	Downregulates *HSP70*, *TGF-β*, *p53*, *Chop*, *Bip*; alleviates brain pathology	Nile tilapia (*Oreochromis niloticus*)	[94]

### 5.5. Cross-Species Synthesis and Methodological Limitations

Viewed collectively, the evidence across the four animal groups reveals recurring mechanistic patterns alongside shared methodological shortcomings. At the mechanistic level, methyl donors (betaine, methionine, folate) consistently show potential for modulating HSP70 expression across poultry, swine, and to a lesser extent ruminants, suggesting that one-carbon metabolism is a conserved pathway for epigenetic regulation of heat stress responses. Similarly, the HDAC-inhibitory properties of SCFAs, particularly butyrate, are well-established in cell models and appear to translate to enhanced gut barrier function across species, although direct evidence in livestock remains circumstantial.

However, significant methodological limitations undermine the robustness of the current evidence base. The most critical weakness is the near-absence of direct epigenetic endpoint measurements—no study cited in this review measured DNA methylation, histone acetylation, or miRNA profiles in livestock under heat stress in conjunction with nutritional interventions. Instead, most conclusions are inferred from changes in mRNA or protein levels, which, while suggestive, do not constitute proof of epigenetic regulation. Furthermore, experimental designs vary widely in terms of stress protocols (duration, intensity), nutritional dosages, and tissue sampling, complicating cross-study comparisons. Addressing these shortcomings in the existing literature will require standardised experimental protocols and, above all, a commitment to measuring epigenetic endpoints directly in future studies—without which the field will struggle to move beyond correlation towards causation.

## 6. Challenges in the Field and Future Research Directions

Although the existing evidence seems promising, translating nutritional epigenetics into reliable farm-level strategies faces obstacles that extend beyond the methodological shortcomings of individual studies. These include unresolved questions about transgenerational inheritance, inter-organ crosstalk, the conservation of epigenetic pathways across species, and the practical challenges of implementing epigenetic biomarkers and nutrient combinations in commercial production systems.

A major challenge is to understand how the epigenetic marks induced by HS are transmitted across generations. Although there is evidence consistent with the hypothesis that epigenetic changes in germ cells can be passed on to offspring and may even pre-adapt them to HS, many aspects remain largely unclear. These include which heritable marks are involved, their stability across generations, and the extent to which nutrition can actively reprogram these epigenetic landscapes in farmed animals [16,100]. Additionally, the epigenetic basis of inter-organ “cross-talk” is not well-defined. HS elicits a systemic response, but how metabolites or exosomal miRNAs originating from the intestine affect the epigenetic regulation of distant organs such as the liver or mammary gland is currently only speculative. Unraveling this inter-organ communication mechanism is crucial for formulating holistic nutritional strategies, although the underlying epigenetic basis remains largely unexplored in livestock [12,101,102]. Another significant challenge is the combination of evolutionary conservation and species-specificity in epigenetic pathways. Due to differences in physiology and metabolism among species, results obtained from model organisms cannot be directly applied to farmed animals. Therefore, detailed mapping of species-specific epigenetic landscapes is required to enable effective interventions [103,104].

Methodologically, a complication in this field is the epigenetic heterogeneity within whole tissues, which masks key cell-type-specific responses. Emerging single-cell multi-omics technologies (such as scATAC-seq, scNMT-seq) hold promise in precisely identifying the cellular targets of nutritional interventions [105,106,107]. Moreover, the development of dynamic monitoring tools is urgently needed. The epigenome is highly plastic, yet most studies rely only on static data from a single time point. Innovations in non-invasive sampling techniques, combined with longitudinal multi-omics data integration, are required to construct a dynamic “nutrition–epigenome–phenotype” axis model [108,109,110,111].

Beyond methodological challenges, another layer of complexity, largely overlooked by current nutritional epigenetics, is the potential for synergistic or antagonistic interactions among dietary components when administered concomitantly. Most livestock studies examine single nutrients in isolation, whereas practical diets consist of complex mixtures in which one compound may enhance, neutralize, or even reverse the epigenetic effect of another. A well-documented example of synergy is the combination of butyrate and curcumin [51,112]. Butyrate, a potent HDAC inhibitor produced by gut microbial fermentation [66,67], has been indicated to enhance the blood–brain barrier permeability of curcumin, leading to synergistic restoration of Wnt/β-catenin pathway antagonists in glioblastoma cells [67,112]. Whether such synergy exists between butyrate and polyphenols in the livestock gut epithelium under HS remains unknown. Nevertheless, given the increasing use of both classes of compounds in feed formulations [113], the possibility is intriguing. Similarly, the gut microbiota itself functions as a metabolic “hub” that can transform dietary polyphenols into bioactive metabolites, which then collaborate with microbial SCFAs to jointly shape the host epigenome. This three-way interaction—diet, microbiome, epigenome—creates both opportunities for synergy and risks of unpredictable outcomes [114].

Conversely, synergistic interactions are not always beneficial, and antagonistic effects are understudied [50]. High concentrations of butyrate, for instance, can accelerate vascular calcification via HDAC-dependent activation of NF-κB signalling, serving as a cautionary example that even “protective” nutrients may exhibit dose-dependent adverse effects [114]. Furthermore, methyl donors and polyphenols may compete for the same metabolic pathways. Excessive methionine or folate supplementation can theoretically overburden one-carbon metabolism, potentially interfering with the bioavailability or efficacy of co-administered phytochemicals [50]. Such interactions have been characterised in cancer cell models but have received no systematic investigation in livestock under HS [115,116]. These observations strongly advocate for moving beyond single-nutrient studies toward rational combination strategies paired with dose–response assessments. This objective aligns seamlessly with the emerging vision of data-driven precision nutrition platforms, which represents a promising but as-yet-unproven direction for livestock production.

**Figure 3:**
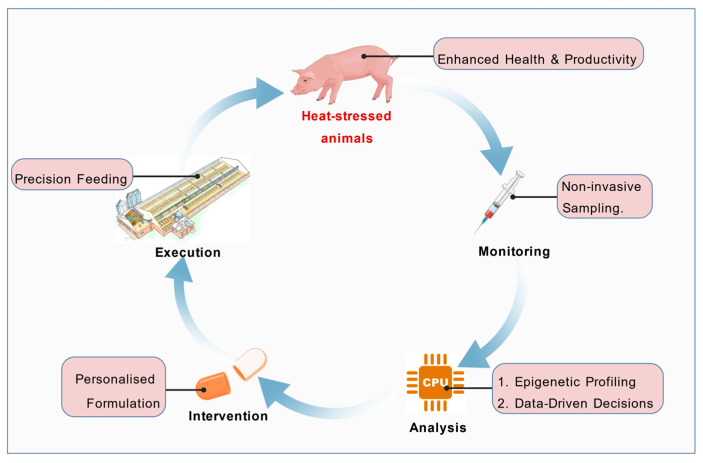
A future perspective on precision nutrition based on epigenetic biomarkers. Arrows indicate the proposed flow from epigenetic biomarker detection to individualized dietary adjustment and improved heat-stress resilience.

Finally, in terms of translational applications, the prospect lies in precision nutrition based on the discovery of epigenetic biomarkers. Conceptually, identifying accessible epigenetic features associated with heat-stress resistance would be transformative, enabling real-time monitoring of animals and proactive dietary management (Figure 3), although the predictive value of such biomarkers in livestock remains to be validated [117,118,119]. Integrating these biomarkers with other data streams into intelligent decision-support platforms would facilitate the development of dynamic, individualized feeding programs, rather than a one-size-fits-all approach [120,121,122,123]. If realised, this vision elevates nutrition from a supportive role to a powerful operational biotechnological tool for enhancing climate resilience.

While precision nutrition based on epigenetic principles holds considerable promise, several practical obstacles must be acknowledged. Cost-effectiveness remains a primary concern: the economic benefit of supplementing methyl donors, SCFAs, or vitamins must clearly outweigh the losses incurred by heat stress, and this balance varies with production system, region, and market conditions. Regulatory pathways for feed additives differ across countries and can impose substantial time and financial costs before a product reaches the market. Production system variability—between intensive, semi-intensive, and extensive operations—further complicates the translation of research findings, as results obtained under controlled experimental conditions may not replicate in commercial settings. Environmental interactions, including the modulating effects of humidity, ventilation, and diet composition, add another layer of complexity that is rarely captured in controlled studies. Finally, the feasibility of epigenetic biomarker monitoring on farms remains unproven: collecting, storing, and analysing blood methylation patterns or salivary miRNA profiles under field conditions poses logistical, technical, and economic challenges that are not easily overcome. These factors should temper expectations and steer future research towards solutions that are both mechanistically sound and practically feasible.

The gaps identified above point to several clear priorities for future work. First and foremost, future studies must move beyond indirect inferences by incorporating direct epigenetic endpoint measurements—bisulfite sequencing for DNA methylation, ChIP-qPCR for histone modifications, and small RNA sequencing for miRNA profiles—to definitively establish whether nutritional interventions truly alter the epigenome in heat-stressed livestock (i). Equally important are systematic dose–response and combination studies that characterise synergistic or antagonistic interactions among methyl donors, SCFAs, vitamins, and phytochemicals, as practical diets contain complex mixtures rather than single nutrients (ii). The field also urgently needs cross-species comparative epigenomics to determine which mechanisms are conserved across poultry, ruminants, swine, and aquatic species, and which are species-specific—a distinction critical for translating findings into species-appropriate feeding strategies (iii). Parallel efforts should focus on validating accessible epigenetic biomarkers, such as methylation patterns in blood or miRNA profiles in saliva, that could enable real-time monitoring of stress resilience and intervention efficacy under commercial conditions (iv). Finally, the tripartite interaction between gut microbiota, host epigenome, and dietary components represents a frontier that is conceptually compelling but experimentally unexplored in livestock; integrating these three axes would provide a more holistic understanding of how nutrition programmes thermal tolerance (v). Collectively, these priorities call for interdisciplinary collaboration between animal nutritionists, epigeneticists, and microbiologists to translate mechanistic insights into practical applications.

## 7. Conclusions

This review integrates compelling evidence of the multi-systemic impacts of HS on farmed animals, with epigenetic mechanisms at the core of the interaction between the environment and genes. We elaborated in detail on how HS dynamically alters DNA methylation, histone modifications, and non-coding RNA regulation. Additionally, we evaluated several nutritional intervention measures, which are associated with changes in the epigenetic state that may contribute to enhanced heat tolerance. It is noteworthy that although the epigenetic patterns of heat response in aquatic animals have been mapped in considerable detail, research on how nutrition regulates heat-induced epigenetic changes remains scarce. Moreover, in the nutrition of farmed animals, the potential synergistic or antagonistic interactions among dietary components are crucial for formulating effective nutritional strategies, yet current research in this area is indeed limited. Looking ahead, it is essential to overcome the related challenges of trans-generational inheritance, inter-organ crosstalk, and species-specificity. Harnessing the plasticity of the epigenome through precision nutrition offers a non-invasive approach to alleviating HS. Resolving these issues will enable the farmed animal industry to proactively build resilience, ensuring sustainable production in the context of climate change. From a practical standpoint, the evidence reviewed here supports the development of dietary formulations that incorporate methyl donors, SCFAs, or specific vitamins during anticipated heat events to help mitigate production losses. More broadly, the mechanistic framework outlined in this review provides a basis for designing precision nutrition strategies that are tailored to species, production systems, and environmental conditions. Such approaches have the potential to move beyond generic recommendations towards targeted, feed-based interventions that strengthen climate resilience in livestock production.

## Figures and Tables

**Table 1:** Modes of action of nutritional compounds on epigenetic machinery.

Mode of Action	Nutrient/Bioactive Compound	Molecular Target/Pathway	Epigenetic Outcome & Potential Functional Role	Primary Evidence Source	Refs.
Enzyme substrate provision	Betaine, Methionine, Choline, Folate, Vitamin B12	One-carbon metabolism; DNMTs	Provides methyl groups for DNA and histone methylation, crucial for stress-responsive gene silencing/activation.	Livestock (indirect); cell models	[51,52,81]
Enzyme inhibition	Butyrate, Propionate, Valerate	HDACs (Class I/IIa)	Global increase in histone acetylation; promotes genes for barrier function, anti-inflammation.	Cell models; inferred in livestock	[53,77,78,79]
	EGCG	DNMT1	Site-specific DNA hypomethylation; reactivates silenced genes.	Cell models; human studies	[68,69]
	Curcumin	HAT (p300/CBP), DNMT1	Modulates both histone acetylation and DNA methylation; exerts anti-inflammatory effects.	Cell models	[70,72]
	Pterostilbene	HDACs (SIRT1), Nrf2 signaling	Promotes histone deacetylation via SIRT1 and can reverse promoter hypermethylation of antioxidant genes.	Cell models	[70,72]
Receptor-mediated signalling	Vitamin D (Calcitriol)	VDR/RXR heterodimer	Recruits HATs to target gene promoters; promotes anti-inflammatory transcriptome.	Cell models; human; inferred in livestock	[62,63,64]
Cofactor provision	α-Ketoglutarate (α-KG)	TET enzymes, JmjC-domain histone demethylases (KDMs)	Essential cofactor for DNA and histone demethylation; promotes epigenetic plasticity.	Cell models	[54,55]
	Zinc (Zn)	Sirtuins (Class III HDACs), ZnF proteins	Cofactor for enzymatic activity and structural integrity of epigenetic complexes.	Cell models; inferred in livestock	[66,67]
	NAD^+^	Sirtuins (Class III HDACs)	Essential co-substrate for sirtuin deacetylase activity; links cellular energy status to epigenetics.	Cell models	[67]
Redox balance & indirect modulation	Selenium, Vitamin E	Glutathione peroxidase, other antioxidant systems	Reduces ROS, preventing oxidative stress-induced global DNA hypomethylation and aberrant histone marks.	Livestock (antioxidant effects); epigenetic inference	[56,60,61]
	Arginine	DNMTs, HDACs (via S-nitrosylation)	NO can directly modify and alter the activity of epigenetic enzymes.	Cell models	[74,75,76]
	Polyphenols	Nrf2/ARE pathway, SIRT1	Indirectly modulates epigenome by activating antioxidant and longevity pathways.	Cell models; human	[70,72]
Microbiome-mediated modulation	Resistant Starch, Dietary Fibre	Gut Microbiota, SCFA production	Increases endogenous production of HDACi.	Rodent models; inferred in livestock	[53,77]
	Probiotics (e.g., *L. rhamnosus*)	Gut–Brain; Gut–Liver Axis	Shown to alter DNA methylation in host brain and gut tissues in model organisms.	Zebrafish/rodent models	[80,82]

## Data Availability

No new data were created or analyzed in this study. Data sharing is not applicable to this article.

## Abbreviations

The following abbreviations are used in this manuscript:

ChIP	chromatin immunoprecipitation
DNMT	DNA methyltransferase
DNMT1	DNA methyltransferase 1
EGCG	Epigallocatechin gallate
HAT	Histone acetyltransferase
H3K4me3	Trimethylation of lysine 4 on histone H3
H3K27ac	Acetylation of lysine 27 on histone H3
HDAC	Histone deacetylase
HPA	Hypothalamic–pituitary–adrenal
HS	Heat stress
HSP	Heat shock protein
IGF-1	Insulin-like growth factor 1
IL-1β	Interleukin-1 beta
LPS	Lipopolysaccharide
miRNA	MicroRNA
NF-κB	Nuclear factor kappa B
NO	Nitric oxide
ROS	Reactive oxygen species
RXR	Retinoid X receptor
SAM	S-adenosylmethionine
SCFA	Short-chain fatty acid
VDR	Vitamin D receptor
WGBS	Whole-genome bisulfite sequencing
ZnF	Zinc finger
ZO-1	Zonula occludens-1
